# Systematic Review and Narrative Synthesis of Economic Evaluations of Prostate Cancer Diagnostic Pathways Incorporating Prebiopsy Magnetic Resonance Imaging

**DOI:** 10.1016/j.euros.2023.03.010

**Published:** 2023-05-05

**Authors:** Samuel W.D. Merriel, Rebekah Hall, Fiona M. Walter, Willie Hamilton, Anne E. Spencer

**Affiliations:** a University of Manchester, Manchester, UK; b University of Exeter, Exeter, UK; c Queen Mary University of London, London, UK

**Keywords:** Prostate cancer, Magnetic resonance imaging, Diagnostic pathway, Economic evaluation, Health economics

## Abstract

**Context:**

Prebiopsy magnetic resonance imaging (MRI) of the prostate has been shown to increase the accuracy of the diagnosis of clinically significant prostate cancer. However, evidence is still evolving about how best to integrate prebiopsy MRI into the diagnostic pathway and for which patients, and whether MRI-based pathways are cost effective.

**Objective:**

This systematic review aimed to assess the evidence for the cost effectiveness of prebiopsy MRI-based prostate cancer diagnostic pathways.

**Evidence acquisition:**

INTERTASC search strategies were adapted and combined with terms for prostate cancer and MRI, and used to search a wide range of databases and registries covering medicine, allied health, clinical trials, and health economics. No limits were set on country, setting, or publication year. Included studies were full economic evaluations of prostate cancer diagnostic pathways with at least one strategy including prebiopsy MRI. Model-based studies were assessed using the Philips framework, and trial-based studies were assessed using the Critical Appraisal Skills Programme checklist.

**Evidence synthesis:**

A total of 6593 records were screened after removing duplicates, and eight full-text papers, reporting on seven studies (two model based) were included in this review. Included studies were judged to have a low-to-moderate risk of bias. All studies reported cost-effectiveness analyses based in high-income countries but had significant heterogeneity in diagnostic strategies, patient populations, treatment strategies, and model characteristics. Prebiopsy MRI-based pathways were cost effective compared with pathways relying on ultrasound-guided biopsy in all eight studies.

**Conclusions:**

Incorporation of prebiopsy MRI into prostate cancer diagnostic pathways is likely to be more cost effective in than that into pathways relying on prostate-specific antigen and ultrasound-guided biopsy. The optimal prostate cancer diagnostic pathway design and method of integrating prebiopsy MRI are not yet known. Variations between health care systems and diagnostic approaches necessitate further evaluation for a particular country or setting to know how best to apply prebiopsy MRI.

**Patient summary:**

In this report, we looked at studies that measured the health care costs and benefits and harms to patients of using prostate magnetic resonance imaging (MRI), to decide whether men need a prostate biopsy for possible prostate cancer. We found that using prostate MRI before biopsy is likely to be less costly for health care services and probably has better outcomes for patients being investigated for prostate cancer. It is still unclear what the best way to use prostate MRI is.

## Take Home Message

Integration of prebiopsy magnetic resonance imaging (MRI) into prostate cancer diagnostic pathways is likely to be more cost effective than traditional pathways employing ultrasound-guided biopsy. It is unclear what the optimal prostate cancer diagnostic pathway design is and how to best integrate prebiopsy MRI.

## 1 Introduction

Prostate cancer incidence has risen globally in recent decades and is expected to continue to rise in many countries [1]. Whilst prostate cancer causes a significant number of cancer-related deaths [2], an increasing proportion of men diagnosed with prostate cancer have low-risk disease that is unlikely to cause significant morbidity or mortality (particularly in men diagnosed at an older age). This is suspected to have been driven in part by the increasing use of prostate-specific antigen (PSA) in clinical practice for screening or early detection for symptomatic men in primary care. However, PSA does not easily distinguish between clinically significant and clinically nonsignificant prostate cancer [3].

Magnetic resonance imaging (MRI) has emerged in recent years as a new diagnostic test for prostate cancer. The two main MRI techniques to assess for prostate cancer are multiparametric MRI (mpMRI), which includes intravenous contrast, and biparametric MRI (bpMRI), which does not require contrast [4]. The PROMIS trial found that using mpMRI scans prior to a prostate biopsy increased the number of men being diagnosed with clinically significant prostate cancer, without increasing overdiagnosis. Furthermore, it could potentially avoid the need for a biopsy altogether in up to 27% of men [5]. A 2019 Cochrane review of all existing clinical evidence suggests that MRI-based prostate cancer diagnostic pathways can result in more accurate diagnoses of clinically significant tumours [6].

The overall annual economic costs of prostate cancer in the UK alone, taking into account health care costs and lost earnings after premature death, has been estimated to be £666 million [7]. In addition to the survival benefits of diagnosing patients with prostate cancer at an earlier stage [8], there are also much lower costs associated with treating a patient with localised prostate cancer [9]. Reductions in the number of men undergoing prostate biopsies for suspected prostate cancer by undergoing MRI of the prostate first could reduce the health care costs associated with the procedure and postbiopsy infections [10]. However, concerns have been raised that the cost of MRI scanners; the availability of, and training requirements for, radiographers and radiologists to perform and interpret images to sufficient quality standards; and the time taken per test could limit the benefits of MRI for prostate cancer–related health care expenditure [4].

The evidence for the cost effectiveness and optimal design of new MRI-based prostate cancer diagnostic pathways is still evolving. A National Institute for Health Research (NIHR) Health Technology Assessment (HTA) report from the PROMIS trial concluded that “incorporating mpMRI into the diagnostic pathway as an initial test prior to prostate biopsy may increase the cost effectiveness of the prostate cancer diagnostic and therapeutic pathway” [11]. There has been no systematic review of economic evaluations that identifies and critically appraises existing evidence for this clinical area. This systematic review aims to assess the evidence for the cost effectiveness of prebiopsy MRI-based prostate cancer diagnostic pathways.

## 2 Evidence acquisition

This systematic review of full economic evaluations (EEs) was guided by a recently published framework developed by van Mastrigt et al [12] and others [13], [14].

### 2.1 Data sources

Bibliographic databases and other sources of publications that were searched included MEDLINE, PubMed, the Cochrane Library, EMBASE, Psycinfo, Cumulative Index of Nursing and Allied Health Literature (CINAHL), Web of Science (WoS), EconLit, clinicaltrials.gov, University of York Centre for Reviews and Dissemination (CRD) database (including NHS Economic Evaluation Database), and the International Standard Randomised Controlled Trial Number registry.

### 2.2 Search strategy

Recommended search strategies for EEs [15] from the INTERTASC Information Specialists Sub-Group (https://sites.google.com/a/york.ac.uk/issg-search-filters-resource/home) for MedLine, EMBASE, PsycINFO, and CINAHL were adapted and combined with subject-specific search terms. Search terms and MeSH headings included MRI OR mpMRI OR “Magnetic Resonance Imaging” OR “Multiparametric MRI” OR “Multiparametric magnetic resonance imaging OR bpMRI OR “Biparametric MRI” AND prostate AND cancer OR malignancy OR neoplas$ OR tumour OR adenocarcinoma AND cost OR “cost effectiveness” OR “health economics” OR economics (see the Supplementary material for full search strategies). Technical reports relating to prostate cancer were also searched for on the National Institute for Health and Care Excellence (NICE) website. References were also obtained by hand searching for relevant papers in the bibliographies of papers and reviews selected. Citation searching was performed via WoS using a search strategy combining the terms prostate and MRI with a proven WoS filter for EEs used in an NIHR HTA by Snowsill et al [16].

### 2.3 Inclusion criteria

Search hits were included in this review if these met the following criteria:

1. Full EEs
2. Assessing prostate cancer diagnostic pathways for adult males that included mpMRI and/or bpMRI as a diagnostic test for prostate cancer prior to biopsy

### 2.4 Exclusion criteria

Search hits were excluded from the study if these met any of the following criteria:

1. Partial EEs
2. Studies that include only diagnostic tests/pathways for prostate cancer that do not feature prebiopsy MRI
3. Case studies
4. Unpublished/incomplete studies
5. Conference abstracts
6. Studies and papers published in languages other than English

No restrictions were placed on publication year, study setting, country, or comparators used. EEs on the basis of modelling and/or randomised controlled trials were considered for inclusion.

### 2.5 Screening search hits

Search hits from each database were downloaded and combined into a review database managed in a shared folder in Mendeley Desktop (version 1.19.4; Mendeley Ltd, New York, NY, USA). An initial search of all identified databases using the proposed search terms was conducted to identify potentially relevant papers through titles and abstracts. Any duplicate search hits were removed. Titles and abstracts of potentially relevant papers were screened by two reviewers (S.W.D.M. and R.H.) independently, using the inclusion/exclusion criteria. In the event of disagreement between reviewers of study eligibility on the basis of title and abstract, a decision was reached by consensus with a third reviewer (W.H.). Full-paper review of all studies included on initial screening of title and abstract was performed by the same two reviewers (S.W.D.M. and R.H.) independently, with any disagreements resolved through discussion with a third reviewer (W.H.).

### 2.6 Data extraction

One reviewer (S.W.D.M.) extracted data from included papers selected using a standardised form. This form was piloted on two included papers and then adapted through an iterative process, before being used for extracting data on the remaining papers. A random selection of 10% of included full-text papers was reviewed by another reviewer (R.H.) to confirm accuracy of data extraction. Disagreements were resolved by consensus discussion, involving a third reviewer (W.H. or A.S.).

Basic study and methods data (first author, year of publication, country, study population, setting, EE type, analytic/modelling approach, time horizon, data sources, currency, discounting, and methods to address uncertainty) were extracted from each included paper. Modelled pathway characteristics were extracted including items such as patient selection criteria, tests done, MRI approach (mpMRI or bpMRI), thresholds for diagnostic test, and non-MRI pathways used for comparison. Primary and secondary outcome measures, including cost effectiveness measures in terms of incremental cost-effectiveness ratios or quality-adjusted life years, were also extracted from each study. The specified contact author of primary studies was contacted in the event that additional data were required for the analysis.

### 2.7 Risk of bias assessment

Assessment of the risk of bias of model-based studies in this review was performed using the framework of Philips et al [17]. The quality assessment of included EEs associated with trials was performed using the Critical Appraisal Skills Programme (CASP) checklist for EE [18].

### 2.8 Narrative synthesis

A narrative synthesis of data extracted from included studies was undertaken. This included a summary of the modelled pathways to compare and contrast different pathways assessed within and between studies. The cost-effectiveness measures were presented and summarised. Data were presented in tabular format and with a hierarchical decision matrix [19].

### 2.9 PRISMA reporting guidelines

This manuscript follows the Preferred Reporting Items for Systematic Reviews and Meta-analyses (PRISMA) statement [20].

### 2.10 Protocol publication

The protocol for this systematic review has been published on PROSPERO (CRD42020182573).

## 3 Evidence synthesis

### 3.1 Searches

**Fig. 1:**
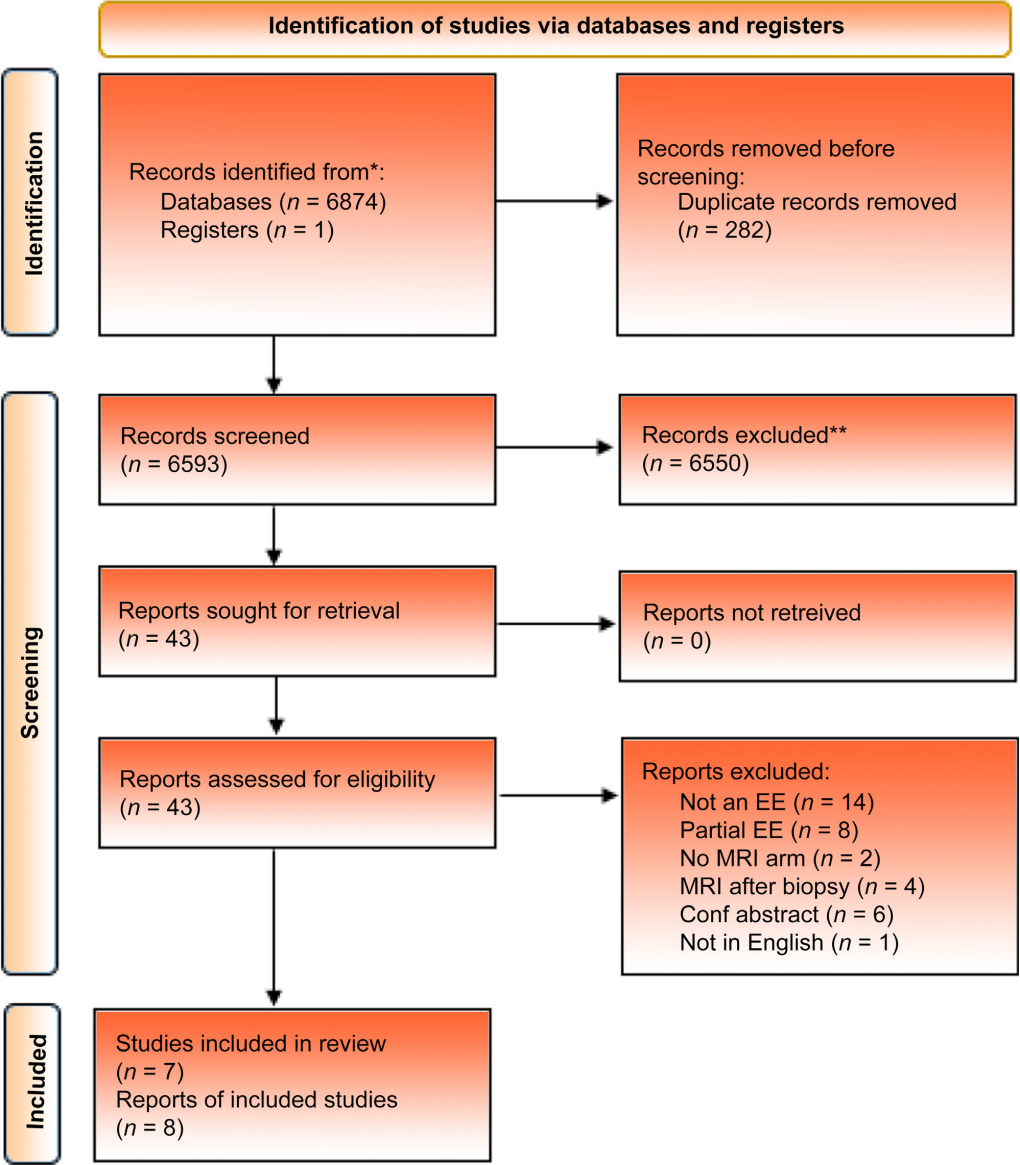
A 2020 PRISMA diagram outlining the number of studies identified, screened, and included in this systematic review. Conf = conference; EE = economic evaluation; MRI = magnetic resonance imaging; PRISMA = Preferred Reporting Items for Systematic Reviews and Meta-analyses.

Database searching yielded 6875 total search hits, with one additional potentially relevant study identified from searching the NICE website. No additional studies were identified through hand searching of reference lists of included papers. After removing duplicates, 6550 studies were excluded on the basis of title and abstract. Forty-three full-text papers were reviewed, and eight studies met the inclusion criteria for this review (see Fig. 1).

### 3.2 Study quality

Two EEs were based on the PROMIS trial [11], [21]. Both studies were assessed as having an overall low risk of bias, with the study by Faria et al [21] meeting all domains of the CASP checklist, with the exception of an incremental analysis (see Table 1). The remaining six studies were model-based EEs. All these had some concerns about study quality in at least four of the 22 elements; most areas of concern fell into the “Structure” domain of the framework from Philips et al [17]. The paper by Pahwa et al [22] was adjudged to have a high risk of bias in two of the nine elements of this domain (see Table 2).

**Table 1:** Quality assessment of trial-based economic evaluations using the CASP checklist [18]

CASP = Critical Appraisal Skills Programme.

Green indicates a low risk of bias, yellow some risk of bias, and red a high risk of bias.

**Table 2:** Quality assessment of model-based economic evaluations using the Philips et al [17] framework

Green indicates a low risk of bias, yellow some risk of bias, and red a high risk of bias.

### 3.3 Study characteristics

The study characteristics can be found in Table 3. Seven studies were published in peer-reviewed journals, and one was an NIHR HTA report. All studies were performed as cost-effectiveness analyses. All were based in a single country, spread across a range of high-income countries with different health care service structures and funding models (USA [*n* = 3], UK [*n* = 2], Canada [*n* = 1], the Netherlands [*n* = 1], and Australia [*n* = 1]). Five studies were conducted in a secondary care setting [11], [21], [23], [24], [25], two were conducted within a national prostate cancer screening programme [26], [27], and one did not state the health care setting within which an MRI-based prostate cancer diagnostic pathway would be utilised [22].

**Table 3:** Study characteristics

Author	Year	Publication type	Study type	Country	Currency	Patient population	Setting
Barnett [27]	2018	Journal article	CEA	USA	USD	Biopsy-naïve men aged 55–69 yr undergoing PSA screening	National PSA Screening Programme
Barnett [26]	2019	Journal article	CEA	USA	USD	Biopsy-naïve men aged 55–69 yr undergoing PSA screening	National PSA Screening Programme
Brown [11]	2018	HTA	CEA	UK	GBP	Men with suspected prostate cancer referred to secondary care	Secondary care
Cerantola [25]	2016	Journal article	CEA	Canada	CAD	Caucasian males aged 60–65 yr with PSA 4–10 ng/ml and life expectancy of 20 yr	Secondary care
de Rooij [23]	2014	Journal article	CEA	The Netherlands	Euros	Average population of men with a suspicion of PCa	Secondary care
Faria [21]	2018	Journal article	CEA	UK	GBP	Men at risk of prostate cancer referred to secondary care	Secondary care
Gordon [24]	2017	Journal article	CEA	Australia	AUD	Australian men aged 60 yr with suspected PCa	Hospital in a public healthcare system
Pahwa [22]	2017	Journal article	CEA	USA	USD	Men aged 41–70 yr	Not stated

AUD = Australian dollars; CAD = Canadian dollars; CEA = cost-effectiveness analysis; GBP = British pounds; HTA = health technology assessment; PCa = prostate cancer; PSA = prostate-specific antigen; USD = US dollars.

The patient populations varied widely. Five studies specified age ranges for men, from age 60 [24] to 41–70 yr [22]. Four studies specified males needed to have a clinical suspicion of prostate cancer [11,^21^,^23^,24], and only Cerantola et al [25] included any criteria about life expectancy (at least 20 yr).

### 3.4 Modelled pathways

Data extracted regarding the diagnostic pathways modelled in the studies is presented in Table 4. All studies, except that by Faria et al [21], specified the clinical criteria for prebiopsy prostate MRI as either a raised PSA or abnormal digital rectal examination (DRE) of the prostate. PSA thresholds were mixed; three studies had a PSA threshold of >4 ng/ml [23], [26], [27], one study limited PSA levels to 4–10 ng/ml [25], one study used age-standardised reference ranges [11], and two studies referred to “abnormal” PSA only [22], [24]. Only the 2018 study by Barnett et al [27] considered symptoms of a possible prostate cancer, in combination with a PSA level of >3 ng/ml, as MRI referral criteria.

**Table 4:** Modelled diagnostic pathways

Author	Year	MRI criteria	MRI approach	MRI reporting	Clinically significant PCa definition	Testing strategies	Treatment strategies
Barnett [27]	2018	PSA >4 ng/ml Or PSA >3 ng/ml + symptoms	Multiparametric	PIRADS v1	Gleason score ≥7	1. TRUSGB 2. mpMRI: (a) PIRADS 3–5 → targeted biopsy, PIRADS 1–2 → TRUSGB (b) PIRADS 4–5 → targeted biopsy, PIRADS 1–3 → TRUSGB 3. mpMRI: (a) PIRADS 3–5 → targeted biopsy, PIRADS 1–2 → no biopsy (b) PIRADS 4–5 → targeted biopsy, PIRADS 1–3 → no biopsy 4. mpMRI: (a) PIRADS 3–5 → combined biopsy, PIRADS 1–2 → TRUSGB (b) PIRADS 4–5 → combined biopsy, PIRADS 1–3 → TRUSGB 5. mpMRI: (a) PIRADS 3–5 → combined biopsy, PIRADS 1–2 → no biopsy. (b) PIRADS 4–5 → combined biopsy, PIRADS 1–3 → no biopsy	Gleason ≥7 → radical prostatectomy Patients aged 80+ yr → WW Gleason 3 + 3 → 48.5% had AS, 51.5% had prostatectomy AS = annual PSA + standard biopsy every 2 yr. Any progression in Gleason score → prostatectomy
Barnett [26]	2019	PSA > 4 ng/ml	Multiparametric ^18^F-choline PET/mpMRI (without DCE)	PIRADS v2 Likert	Gleason score ≥3 + 4	1. TRUSGB 2. mpMRI: Likert 4–5 → combined biopsy, Likert 1–3 → TRUSGB 3. mpMRI: PIRADS v2 3–5 → combined biopsy, PIRADS v2 1–2 → TRUSGB 4. ^18^F-choline PET/mpMRI: Likert 4–5 → combined biopsy, Likert 1–3 → TRUSGB 5. ^18^F-choline PET/mpMRI: PIRADS v2 3–5 → combined biopsy, PIRADS v2 1–2 → TRUSGB 6. mpMRI: Likert 4–5 → combined biopsy, Likert 1–3 → no biopsy 7. mpMRI: PIRADS v2 3–5 → combined biopsy, PIRADS v2 1–2 → no biopsy 8. ^18^F-choline PET/mpMRI: Likert 4–5 → combined biopsy, Likert 1–2 → no biopsy 9. ^18^F-choline PET/mpMRI: PIRADS v2 3–5 → combined biopsy, PIRADS v2 1–2 → no biopsy	Gleason 3 + 4 or higher → Radical prostatectomy Patients aged 80+ yr → WW Gleason 3 + 3 → 48.5% had AS, 51.5% had prostatectomy AS = annual PSA + standard biopsy every 2 yr. Any progression in Gleason score → prostatectomy
Brown [11]	2018	PSA elevated above age-reference standard Or Abnormal DRE	Multiparametric	Likert	Primary—dominant Gleason pattern 4–5 and/or cancer core length >6 mm Secondary—any Gleason 4+ and/or cancer core length >4 mm	32 diagnostic strategies using mpMRI, TRUSGB, and TPMB in different combinations, for each of the two diagnostic definitions for mpMRI, TRUSGB, and TPMB, and between two and five cut-off points on the mpMRI Likert scale for suspicion of cancer, under the following principles: 1. The only tests considered are mpMRI, TRUSGB and TPMB. This follows from PROMIS, which compared mpMRI and TRUSGB with TPMB. 2. There can be up to three tests in one diagnostic strategy. Diagnostic episodes may be repeated over time, but this is not explicitly modelled in this analysis. 3. A diagnostic strategy can include up to two biopsies. 4. If included in the strategy, mpMRI can be used only once.	Low-risk cancer → AS Intermediate-risk cancer → AS or radical treatment High-risk cancer → AS or radical treatment
Cerantola [25]	2016	Abnormal DRE Or PSA 4-10 ng/ml	Multiparametric	PIRADS v1	Not stated	1. MRTB strategy: Positive MRI → MRTB Positive MRTB → treatment Negative MRTB → follow-up as required Negative MRI → follow-up as required 2. TRUSGB strategy: Positive TRUSGB → treatment Negative TRUSGB → follow-up as required	Distributed to AS or definitive treatment based on risk stratification at diagnosis After AS or initial treatment, patients could die, relapse, or progress to CRPC
de Rooij [23]	2014	PSA >4 ng/ml and suspicion of PCa	Multiparametric	Radiologist report	Large Gleason score 3 + 3 tumour Or Gleason score ≥3 + 4	1. MRI strategy = mpMRI for all men, with MRGB for +ve mpMRI 2. TRUSGB strategy = TRUS for all men	Radical prostatectomy Radiation therapy Brachytherapy WW/AS (Probabilities of receiving treatment for patients diagnosed with clinically significant or insignificant tumours derived from the literature and expert opinion)
Faria [21]	2018	Not stated	Multiparametric	Likert	1 Gleason Score ≥ 4+3 or max core length ≥ 6mm 2 Gleason Score ≥ 3+4 or max core length ≥ 4mm	The diagnostic strategies consisted of clinically feasible combinations of MPMRI, TRUSGB, and TPMB, in addition to the use of TRUSGB and TPMB in isolation. A diagnosis of CS cancer requires a biopsy; hence strategies were defined to always end with a confirmatory biopsy. Each of the 32 test combinations were tested for the alternative classifications and cut-offs, returning a total of 383 strategies	Low-risk cancer → WW Intermediate-risk cancer → WW or radical prostatectomy High-risk → radical prostatectomy
Gordon [24]	2017	Abnormal PSA And/or Abnormal DRE	Multiparametric	Radiologist report	Not stated	Prebiopsy mpMRI, followed by TRUSGB, TPUSGB, or MRTB	Population-based proportions of men with PCa receiving treatments: 1. AS for under 75 yr 2. WW for 75 yr and over 3. Radical prostatectomy 4. External beam radiotherapy 5. Brachytherapy 6. Androgen deprivation therapy
Pahwa [22]	2017	Elevated PSA Or Abnormal DRE	Biparametric	Not stated	Gleason score ≤6 and tumour volume <0.5 mm^3^	1. Standard TRUSGB 2. bpMRI + cognitive MR-guided biopsy if MRI +ve 3. bpMRI + fusion-guided biopsy if MRI +ve 4. bpMRI + in-bore MR-guided biopsy if MRI +ve 5. bpMRI + cognitive MR-guided biopsy if MRI +ve or TRUSGB if MRI –ve 6. bpMRI + fusion-guided biopsy if MRI +ve or TRUSGB if MRI –ve 7. bpMRI + in-bore MR-guided biopsy if MRI +ve or TRUSGB if MRI –ve	Probabilities of patient choosing treatment in clinically significant and insignificant cancer derived from the literature Options: 1. AS 2. WW 3. Radiation therapy 4. Brachytherapy 5. Prostatectomy 6. Androgen deprivation therapy

AS = active surveillance; bpMRI = biparametric MRI; CRPC = castration-resistant prostate cancer; CS = clinically significant; DCE = dynamic contrast enhancement; DRE = digital rectal examination; mpMRI = multiparametric MRI; MRGB = magnetic resonance imaging guided biopsy; MRI = magnetic resonance imaging; MRTB = magnetic resonance imaging targeted biopsy; PCa = prostate cancer; PET = positron emission tomography; PIRADS = Prostate Imaging Reporting and Data Systems; PSA = prostate-specific antigen; TPMB = template prostate mapping biopsy; TPUSGB = transperineal ultrasound guided biopsy; TRUS = transrectal ultrasound; TRUSGB = transrectal ultrasound guided biopsy; WW = watchful waiting.

Seven studies specified the use of mpMRI for the detection of prostate cancer. Pahwa et al [22] incorporated bpMRI into the modelled diagnostic pathway and compared it with mpMRI as part of the sensitivity analysis to assess whether the addition of dynamic contract enhancement affected the cost effectiveness of the pathway. MRI reporting was mixed: two studies relied on Prostate Imaging Reporting and Data Systems (PIRADS) version 1 [25], [27], one study used PIRADS version 2 [26], two studies used Likert scales for the likelihood of a lesion being a prostate cancer [11], [21],; and two relied on radiologist reports [23], [24]. Six studies gave definitions for clinically significant prostate cancer, and these were all different (see Table 4).

All studies compared MRI-based pathways with the more traditional route employing transrectal ultrasound (TRUS) biopsy in men with possible prostate cancer, with the exception of Gordon et al [24] in which all men had mpMRI followed by a TRUS biopsy, transperineal ultrasound-guided biopsy, or MRI-guided biopsy to confirm the diagnosis and select the appropriate treatment. The 2019 Barnett et al [26] study compared TRUS biopsy with mpMRI pathways and combined mpMRI/18 F-choline positron emission tomography scanning to assess whether the combined scan was more cost effective for detecting clinically significant prostate cancer than mpMRI alone in the prebiopsy setting. The number of different testing pathways compared within studies ranged from 2 to 383. For the MRI-based pathways, men with a negative MRI result were assumed not to go on for biopsy, and were either discharged at that point or had some further clinical follow-up. A range of different biopsy approaches for men with positive MRI results were modelled.

All studies modelled treatment outcomes on the basis of the diagnoses made in the various modelled testing strategies. Most studies assumed that biopsy results were perfectly accurate for the presence and grade of the prostate cancer. The modelling-based studies estimated the proportion of men opting for different treatment options based on a review of the literature, national prostate cancer registries, and/or expert opinion. The two trial-based EEs based the options for treatment of different prostate cancer risk groups on national guidance but did not explain what proportion of men diagnosed with intermediate- or high-risk prostate cancer received radical treatment, or how this was measured [11], [21].

### 3.5 Model characteristics

The characteristics of the models from the studies are presented in Supplementary Table 1. Three studies combined a decision tree model with a Markov model [11], [21], [23]. Pahwa et al [22] employed a decision-analytic model. Four studies employed lifetime horizon modelling [21], [22], [26], [27], with the remaining time horizons ranging from 10 to 30 yr. Studies outside the USA took a health department or governmental perspective, with two US studies considering MRI-based pathways from a third-payer perspective [26], [27]. Pahwa et al [22] did not state which perspective their study was conducted from. Annual discounting ranged from 1.5% to 5%. All studies performed sensitivity analyses.

### 3.6 Study outcomes

The key outcomes from the modelling studies of prebiopsy MRI for prostate cancer are presented in Table 5. MRI-based pathways were less expensive than TRUS biopsy pathways in six of the studies. MRI-based pathways were found to be more effective in all eight studies. The most cost-effective testing strategies in each study varied in terms of the type of prebiopsy MRI test, threshold for a positive MRI result, and biopsy approach for men with positive MRI. Cerantola et al [25] and de Rooij et al [23] featured the most similar MRI-based pathway, whereby patients with suspected prostate cancer undergo mpMRI, followed by MRI-guided biopsy for men with a positive mpMRI result; the approach is currently in use in the UK. Both studies found the MRI-based pathway to be more effective than a TRUS-biopsy pathway but differed on whether it was more or less expensive. Despite applying slightly different methods to the data generated from the same trial, both Brown et al [11] and Faria et al [21] concluded that the same testing strategy was most cost effective from various combinations of mpMRI, TRUS biopsy, and template prostate mapping biopsy.

**Table 5:** Study outcomes

Author	Year	MRI-based pathway more expensive	MRI-based pathway more effective	Optimal testing strategy	QALYs gained	ICER	Threshold
Barnett [27]	2018	Yes	Yes	mpMRI PIRADS 3–5 → combined biopsy PIRADS 1–2 → no biopsy	60.7 (95% CI 60.1–61.3) QALYs gained compared with no screening	23 483 USD (per 1000 men)	WTP 100 000 USD per QALY
Barnett [26]	2019	No	No	^18^F-choline PET/mpMRI Likert 4–5 → combined biopsy Likert 1–3 → no biopsy	60.4 (95% CI 59.4– 61.4) compared with no screening	35 108 USD (per 1000 men)	WTP 100 000 USD per QALY
Brown [11]	2018	No	Yes	Testing all men with mpMRI at definition 2, cut-off point 2 for CS cancer, using MRTB to detect CS cancer, and rebiopsying men in whom CS cancer was not detected	8.72 (95% CI 8.40–9.04) discounted QALYs gained	Not presented	£13 000, £20 000, or £30 000 per QALY gained
Cerantola [25]	2016	No	Yes	MRTB strategy	0.168 (95% CI not stated) incremental QALYs at 20 yr	Not presented	50 000 CAD per QALY gained
de Rooij [23]	2014	Yes	Yes	MRI strategy	0.10 (95% CI –0.18, 0.34) incremental QALYs at 10 yr	€323	Range of WTP thresholds from €1 to €100 000
Faria [21]	2018	No	Yes	Testing all men with mpMRI at definition 2, cut-off point 2 for CS cancer, using MRTB at definition 2 to detect CS cancer, repeat biopsy for men in whom CS cancer was not detected	8.72 (95% CI 8.40, 9.04)	£7076	£13 000, £20 000, or £30 000 per QALY gained
Gordon [24]	2017	Yes (if same rates of AS) No (if increased rates of AS)	No (if same rates of AS) Yes (if increased rates of AS)	All men receive mpMRI. Positive mpMRI → TRUSGB, TPUSGB, or MRTB. All men with very-low–risk or low-risk cancer assumed to undergo AS	7.83 (95% CI not stated)	3980 AUD	WTP 50 000 AUD per QALY gained
Pahwa [22]	2017	No (except strategy 6)	Yes	bpMRI with cognitive MR-guided biopsy if MRI +ve	8.90 (95% CI 7.34, 10.21) NHB in QALYs	8946 USD	WTP 10 000, 25 000, 50 000, or 100 000 USD

AS = active surveillance; AUD = Australian dollars; CAD = Canadian dollars; CI = confidence interval; CS = clinically significant; ICER = incremental cost effectiveness ratio (cost per QALY gained); mpMRI = multiparametric magnetic resonance imaging; MR = magnetic resonance; MRTB = magnetic resonance imaging targeted biopsy; NHB = net health benefit; PET = positron emission tomography; PIRADS = Prostate Imaging Reporting and Data Systems; QALY = quality adjusted life year; TPUSGB = transperineal ultrasound guided biopsy; TRUSGB = transrectal ultrasound guided biopsy; USD = US dollars; WTP = willingness to pay.

These were the only studies to suggest that a repeat biopsy was needed for patients with positive MRI and a negative initial biopsy, an approach that was still found to be cost effective.

## 4 Conclusions

### 4.1 Key findings

This systematic review of EEs of prostate cancer diagnostic pathways incorporating prebiopsy prostate MRI found significant variation in terms of patient setting, modelled pathways, and key parameters for the included studies. Included EEs were all based in high-income countries where prebiopsy MRI is being implemented to different degrees, with health care systems that are funded in very different ways. Despite the differences between studies, cost-effectiveness outcomes were in favour of MRI-based prostate cancer diagnostic pathways compared with pathways relying on TRUS biopsy.

The studies varied widely in a number of key parameters. The patient populations included either men aged 55–69 yr participating in a prostate cancer screening programme or men with suspected prostate cancer based on elevated PSA or abnormal DRE in different age ranges. A range of MRI reporting systems were modelled, including PIRADS v1, PIRADS v2, and Likert scales. This is an important consideration for diagnostic accuracy of prebiopsy MRI, as each system produces different results [28], [29]. Prostate biopsy approach is another area where there is no clinical consensus for an optimal method [4], and this is reflected in the fact that a number of different biopsy approaches were modelled in the included studies. The definition of clinically significant prostate cancer was also different in each study that reported it, which stems from the fact there is no clear consensus definition at this time [30]. A key purported benefit of MRI in prostate cancer detection is that more patients with clinically significant prostate cancers are diagnosed. This should result in better outcomes for patients and reduced health care costs by reducing overdiagnosis and overtreatment of prostate cancers that are very unlikely to cause significant morbidity or mortality.

There were some areas of commonality among the studies in this systematic review. All studies undertook a cost-effectiveness analysis, with all but one utilising a Markov model. All studies performed sensitivity analyses to varying degrees. Seven of eight studies employed mpMRI, with Pahwa et al [22] using bpMRI. All studies included a TRUS biopsy–only diagnostic pathway for comparison with their proposed MRI-based pathway(s), which was the standard of care before the prostate MRI era.

### 4.2 Comparison with published literature

The only other literature review addressing the question of the cost effectiveness of prebiopsy MRI for prostate cancer detection was undertaken by Chiu and Adcock [31] in a 2018 report compiled for the Canadian Agency for Drugs and Technology in Health. Six studies were included in that review [11], [23], [25], [32], [33], [34], and the authors concluded that MRI prior to TRUS biopsy was more cost effective than TRUS biopsy alone despite higher testing costs. The review by Chiu and Adcock [31] did not cover as wide a range of databases as this review, was only single screened, and was trying to establish the evidence for effectiveness as well as cost effectiveness of prebiopsy MRI. Even so, the conclusions were similar to this review.

Willis et al [35] undertook a literature review of EEs of prostate cancer diagnostic strategies involving imaging, with a particular interest in the evidence for mpMRI. That review considered cost-effectiveness studies of prebiopsy mpMRI and mpMRI performed after an initial negative biopsy where prostate cancer is still suspected. The inclusion criteria were very broad and methods were sparingly reported, although the authors stated that these were available upon request. Five EEs [23], [33], [34], [36], [37] were included in the review by Willis et al [35] of which two studies found diagnostic strategies incorporating mpMRI to be cost effective, two studies did not conclude them to be cost effective, and one study did not find clear evidence either way. The authors judged that existing studies of the cost effectiveness of prostate cancer diagnostic pathways involving MRI lacked consistency in reporting and key modelling assumptions, and that future studies needed broader sensitivity analyses to gain a clearer understanding.

### 4.3 Strengths and limitations

This study has a number of key strengths increasing confidence in the findings. It is the first review addressing the cost effectiveness of prebiopsy MRI for prostate cancer detection conducted in a systematic manner. We adhered closely to the PRISMA guidelines for the conduct of a systematic review, with two independent reviewers screening articles, extracting data, and assessing study quality. A wide range of relevant databases were searched in order to capture a more complete number of studies for possible inclusion. The included studies were generally assessed as having a low risk of bias, improving the likelihood of contributing strong evidence to the review.

However, there are some limitations that need to be taken into consideration when interpreting the findings of this study. The limited number of studies and significant heterogeneity in the conduct, setting, countries, and modelled pathways of the EEs limits comparisons between studies and settings. The health care funding models and systems followed in different countries may also mean that there is no single optimal method for incorporating prebiopsy prostate MRI into diagnostic pathways. MRI is a relatively new test for prostate cancer detection, with new evidence around the optimal use of MRI being published every year. Some key clinical controversies remain unanswered, such as a consensus definition of clinically significant prostate cancer and the optimal biopsy approach for men with suspected prostate cancer, which create variation in the assumptions of cost-effectiveness analyses as demonstrated in the studies for this review. In spite of the differences in key assumptions for cost-effectiveness analyses, the direction of benefit was the same in all studies.

### 4.4 Implications for policy and practice

Prebiopsy prostate MRI for the detection of clinically significant prostate cancer is already recommended in national guidelines in the UK [38], Europe [39], and Australia [40]. The use of prostate MRI in other high-income countries with wider availability of MRI scanners and greater diagnostic workforce capacity to support their use is growing as the evidence for the use of this test evolves. All but one of the studies took the perspective of health care system decision-makers and third-party payers, increasing the relevance of the findings for those deciding whether to fund prostate MRI for their local or national service. This review suggests that commissioning and recommending prebiopsy prostate MRI will result in better outcomes for patients with suspected prostate cancer for the relevant costs involved. Relatively little consideration was given to the opportunity cost of MRI, and the potential for increasing prostate MRIs to reduce availability of MRI scanners and staff resources for other uses of MRI. Health care decision-makers will need to review their available MRI scanners and radiology department workforce to assess the implications of introducing MRI-based prostate cancer pathways on the wider health service.

Most studies in this review modelled mpMRI as the imaging modality of choice. There is growing evidence that bpMRI has equivalent diagnostic accuracy for clinically significant prostate cancer to mpMRI and has the added benefit of a shorter scan time and not requiring the administration of intravenous contracts [41], [42], [43]. As the evidence for bpMRI evolves in years to come, the optimal MRI approach for use in clinical practice may change.

Prostate cancer diagnostic pathways in secondary care or screening programmes that incorporate prebiopsy MRI are likely to be more cost effective than the pathways relying on TRUS biopsy as a diagnostic test alone. Owing to the lack of consensus in a number of areas related to prostate MRI and MRI-guided biopsy techniques, the currently available EEs varied in a number of key parameters. It is unknown what impact the presentation, triage testing, risk stratification, and identification of patients with suspected prostate cancer in primary care for referral to secondary care has on the cost effectiveness of the prostate cancer diagnostic pathway. Further clinical and health economic research is needed to determine the optimal application of prebiopsy prostate MRI to maximise benefits for patients and health care budgets in particular countries and health care systems.

  ***Author contributions*:** Samuel W.D. Merriel had full access to all the data in the study and takes responsibility for the integrity of the data and the accuracy of the data analysis.

  *Study concept and design*: Merriel, Walter, Hamilton, Spencer.

*Acquisition of data*: Merriel.

*Analysis and interpretation of data*: Merriel, Hall.

*Drafting of the manuscript*: Merriel.

*Critical revision of the manuscript for important intellectual content*: Hall, Walter, Hamilton, Spencer.

*Statistical analysis*: Merriel.

*Obtaining funding*: Walter, Hamilton.

*Administrative, technical, or material support*: None.

*Supervision*: Hamilton.

*Other*: None.

  ***Financial disclosures:*** Samuel W.D. Merriel certifies that all conflicts of interest, including specific financial interests and relationships and affiliations relevant to the subject matter or materials discussed in the manuscript (eg, employment/affiliation, grants or funding, consultancies, honoraria, stock ownership or options, expert testimony, royalties, or patents filed, received, or pending), are the following: None.

  ***Funding/Support and role of the sponsor*:** This research arises from the CanTest Collaborative, which is funded by Cancer Research UK (C8640/A23385). The funder had no role in the design and conduct of the study; collection, management, analysis, and interpretation of the data; or preparation, review, or approval of the manuscript.

  ***Data sharing*:** Data are available for bona fide researchers who request it from the authors.

  ***Acknowledgements*:** The authors would like to thank the members of the PPI&E Group that informed this work: Paul Greensmith, Robert Griffin, Fred Miller, Peter Buttle, Alan Cleverley, Dave Butterworth, Nick Taylor, and Tony Wellingham.
