# Supplementary material for: Systematic Review and Narrative Synthesis of Economic Evaluations of Prostate Cancer Diagnostic Pathways Incorporating Prebiopsy Magnetic Resonance Imaging
Source: Eur Urol Open Sci. 2023 May 5;52:123–34. doi: 10.1016/j.euros.2023.03.010 (PMC10193166; doi:10.1016/j.euros.2023.03.010)
Supplement: Supplementary data 2 [file mmc2.docx]

**Full database search strategy and hits**

Medline

| **Search term** | **Hits** |
| --- | --- |
| 1. (prostat* adj3 (cancer* or carcinoma* or malignan* or tumo?r* or neoplas* or adeno*)).af. | 187609 |
| 1. prostatic neoplasms.af. | 128153 |
| 1. exp Prostatic Intraepithelial Neoplasia/ | 1365 |
| 1. exp PROSTATE/ | 35905 |
| 1. or/1-4 | 204278 |
| 1. Economics/ | 27917 |
| 1. exp "costs and cost analysis"/ | 236393 |
| 1. Economics, Dental/ | 1911 |
| 1. exp economics, hospital/ | 24514 |
| 1. Economics, Medical/ | 9077 |
| 1. Economics, Nursing/ | 3999 |
| 1. Economics, Pharmaceutical/ | 2938 |
| 1. (economic$ or cost or costs or costly or costing or price or prices or pricing or pharmacoeconomic$).ti,ab. | 794486 |
| 1. (expenditure$ not energy).ti,ab. | 29812 |
| 1. value for money.ti,ab. | 1692 |
| 1. budget$.ti,ab. | 29253 |
| 1. or/6-16 | 946393 |
| 1. ((energy or oxygen) adj cost).ti,ab. | 4104 |
| 1. (metabolic adj cost).ti,ab. | 1416 |
| 1. ((energy or oxygen) adj expenditure).ti,ab. | 25027 |
| 1. or/18-20 | 29566 |
| 1. 17 not 21 | 939546 |
| 1. letter.pt. | 1087262 |
| 1. editorial.pt. | 533922 |
| 1. historical article.pt. | 358935 |
| 1. or/23-25 | 1960536 |
| 1. 22 not 26 | 903243 |
| 1. exp animals/ not humans/ | 4712329 |
| 1. 27 not 28 | 846074 |
| 1. bmj.jn | 79957 |
| 1. "cochrane database of systematic reviews".jn. | 14876 |
| 1. health technology assessment winchester england.jn. | 1321 |
| 1. or/30-32 | 96154 |
| 1. 29 not 33 | 839828 |
| 1. "biparametric MRI".mp. | 69 |
| 1. bpMRI.mp. | 48 |
| 1. exp Diffusion magnetic resonance imaging/ | 27681 |
| 1. 35 or 36 or 37 | 27757 |
| 1. multiparametric magnetic resonance imaging.mp. | 1386 |
| 1. mpMRI.mp. [mp=title, abstract, original title, name of substance word, subject heading word, floating sub-heading word, keyword heading word, organism supplementary concept word, protocol supplementary concept word, rare disease supplementary concept word, unique identifier, synonyms] | 1041 |
| 1. magnetic resonance imaging.mp. [mp=title, abstract, original title, name of substance word, subject heading word, floating sub-heading word, keyword heading word, organism supplementary concept word, protocol supplementary concept word, rare disease supplementary concept word, unique identifier, synonyms] | 514658 |
| 1. mri.mp. [mp=title, abstract, original title, name of substance word, subject heading word, floating sub-heading word, keyword heading word, organism supplementary concept word, protocol supplementary concept word, rare disease supplementary concept word, unique identifier, synonyms] | 248691 |
| 1. or/39-42 | 572044 |
| 1. 38 or 43 | 575878 |
| 1. 5 and 34 and 44 | 325 |

EMBASE

| **Search term** | **Hits** |
| --- | --- |
| 1. Health Economics.af | 70688 |
| 1. Exp Economic Evaluation/ | 305387 |
| 1. exp Health Care Cost/ | 290365 |
| 1. pharmacoeconomics/ | 7301 |
| 1. 1 or 2 or 3 or 4 | 558324 |
| 1. (econom$ or cost or costs or costly or costing or price or prices or pricing or pharmacoeconomic$).ti,ab. | 1063793 |
| 1. (expenditure$ not energy).ti,ab. | 40291 |
| 1. (value adj2 money).ti,ab. | 2430 |
| 1. budget$.ti,ab. | 38479 |
| 1. 6 or 7 or 8 or 9 | 1099995 |
| 1. 5 or 10 | 1340159 |
| 1. letter.pt. | 1120824 |
| 1. editorial.pt. | 656739 |
| 1. note.pt. | 801764 |
| 1. 12 or 13 or 14 | 2579327 |
| 1. 11 not 15 | 1236647 |
| 1. (metabolic adj cost).ti,ab. | 1507 |
| 1. ((energy or oxygen) adj cost).ti,ab. | 4318 |
| 1. ((energy or oxygen) adj expenditure).ti,ab. | 31676 |
| 1. 17 or 18 or 19 | 36399 |
| 1. 16 not 20 | 1229225 |
| 1. animal/ | 1459554 |
| 1. exp animal experiment/ | 2559977 |
| 1. nonhuman/ | 6213893 |
| 1. (rat or rats or mouse or mice or hamster or hamsters or animal or animals or dog or dogs or cat or cats or bovine or sheep).ti,ab,sh. | 5710948 |
| 1. 22 or 23 or 24 or 25 | 8911462 |
| 1. exp human/ | 21024838 |
| 1. human experiment/ | 500972 |
| 1. 27 or 28 | 21026436 |
| 1. 26 not (26 and 29) | 6511540 |
| 1. 21 not 30 | 1116161 |
| 1. 0959-8146.is. | 61726 |
| 1. 1469-493X or 1366-5278).is. | 22875 |
| 1. 1756-1833.en. | 33354 |
| 1. 32 or 33 or 34 | 106843 |
| 1. 31 not 35 | 1108855 |
| 1. conference abstract.pt. | 3816855 |
| 1. 36 not 37 | 903809 |
| 1. (prostat* adj3 (cancer* or carcinoma* or malignan* or tumo?r* or neoplas* or adeno*)).tw. | 213809 |
| 1. Prostate.sh. | 46299 |
| 1. 39 or 40 | 239670 |
| 1. "multiparametric magnetic resonance imaging".tw. | 1753 |
| 1. mpMRI.tw. | 2353 |
| 1. "magnetic resonance imaging".tw. | 274646 |
| 1. mri.tw. | 414342 |
| 1. "multiparametric mri".tw. | 2511 |
| 1. 42 or 43 or 44 or 45 or 46 | 561594 |
| 1. bpmri.mp. | 58 |
| 1. exp diffusion weighted imaging/ | 41638 |
| 1. "biparametric mri".mp. | 82 |
| 1. "biparametric magnetic resonance imaging".mp. | 46 |
| 1. 48 or 50 or 51 | 118 |
| 1. 47 or 52 | 561595 |
| 1. 47 or 49 or 52 | 577791 |
| 1. 38 and 41 and 54 | 310 |

PsycINFO

| **Search term** | **Hits** |
| --- | --- |
| 1. Prostate.af. | 21759 |
| 1. (prostat* adj3 (cancer* or carcinoma* or malignan* or tumo?r* or neoplas* or adeno*)).af. | 19267 |
| 1. 1 or 2 | 22019 |
| 1. mpMRI.af. | 1 |
| 1. mri.af. | 130514 |
| 1. "multiparametric magnetic resonance imaging".af. | 45 |
| 1. "multiparametric mri".af. | 235 |
| 1. "magnetic resonance imaging".af. | 147502 |
| 1. 4 or 5 or 6 or 7 or 8 | 192087 |
| 1. "costs and cost analysis"/ | 16711 |
| 1. "Cost Containment"/ | 611 |
| 1. (economic adj2 evaluation$).ti,ab. | 1725 |
| 1. (economic adj2 analy$).ti,ab. | 1549 |
| 1. (economic adj2 (study or studies)).ti,ab. | 811 |
| 1. (cost adj2 evaluation$).ti,ab. | 342 |
| 1. (cost adj2 analy$).ti,ab. | 3736 |
| 1. (cost adj2 (study or studies)).ti,ab. | 878 |
| 1. (cost adj2 effective$).ti,ab. | 15432 |
| 1. (cost adj2 benefit$).ti,ab. | 3514 |
| 1. (cost adj2 utili$).ti,ab. | 1280 |
| 1. (cost adj2 minimi$).ti,ab. | 374 |
| 1. (cost adj2 consequence$).ti,ab. | 116 |
| 1. (cost adj2 comparison$).ti,ab. | 188 |
| 1. (cost adj2 identificat$).ti,ab. | 26 |
| 1. (pharmacoeconomic$ or pharmaco-economic$).ti,ab. | 315 |
| 1. Or/10-25 | 35113 |
| 1. (task adj2 cost$).ti,ab,id. | 648 |
| 1. (switch$ adj2 cost$).ti,ab,id. | 1356 |
| 1. (metabolic adj cost).ti,ab,id. | 103 |
| 1. ((energy or oxygen) adj cost).ti,ab,id. | 287 |
| 1. ((energy or oxygen) adj expenditure).ti,ab,id. | 2734 |
| 1. or/27-31 | 4836 |
| 1. (animal or animals or rat or rats or mouse or mice or hamster or hamsters or dog or dogs or cat or cats or bovine or sheep or ovine or pig or pigs).ab,ti,id,de. | 354985 |
| 1. editorial.dt. | 44086 |
| 1. letter.dt. | 22468 |
| 1. dissertation abstract.pt. | 498253 |
| 1. or/33-36 | 897583 |
| 1. (0003-4819 or 0003-9926 or 0959-8146 or 0098-7484 or 0140-6736 or 0028-4793 or 1469-493X).is. | 13343 |
| 1. 26 not (32 or 37 or 38) | 30075 |
| 1. 3 and 9 and 39 | 14 |

Web of Science

| **Search term** | **Hits** |
| --- | --- |
| 1. TOPIC:(prostate) Indexes=SCI-EXPANDED, SSCI, A&HCI, CPCI-S, CPCI-SSH, ESCI Timespan=All years | 275543 |
| 1. TS=(cancer OR malignancy OR neoplas$ OR tumour OR adenocarcinoma) Indexes=SCI-EXPANDED, SSCI, A&HCI, CPCI-S, CPCI-SSH, ESCI Timespan=All years | 3538950 |
| 1. #1 and #2 | 230656 |
| 1. TS=(MRI OR mpMRI OR "Magnetic Resonance Imaging" OR "Multiparametric MRI" OR "Multiparametric magnetic resonance imaging" OR bpMRI OR "Biparametric MRI") Indexes=SCI-EXPANDED, SSCI, A&HCI, CPCI-S, CPCI-SSH, ESCI Timespan=All years | 445352 |
| 1. TS=(economic* or cost or costs or costly or costing or costed or price or prices or pricing or priced or discount or discounts or discounted or discounting or ration* or expenditure or expenditures or budget* or afford* or pharmacoeconomic or pharmaco-economic*) Indexes=SCI-EXPANDED, SSCI, A&HCI, CPCI-S, CPCI-SSH, ESCI Timespan=All years | 3562854 |
| 1. TS=(markov* or monte carlo) Indexes=SCI-EXPANDED, SSCI, A&HCI, CPCI-S, CPCI-SSH, ESCI Timespan=All years | 414269 |
| 1. TS=(decision near/2 (tree* or analy* or model*) ) Indexes=SCI-EXPANDED, SSCI, A&HCI, CPCI-S, CPCI-SSH, ESCI Timespan=All years | 97099 |
| 1. TS=(survival near/3 analys*) Indexes=SCI-EXPANDED, SSCI, A&HCI, CPCI-S, CPCI-SSH, ESCI Timespan=All years | 66737 |
| 1. TS=(qol* or qoly or qolys or hrqol* or qaly or qalys or qale or qales) Indexes=SCI-EXPANDED, SSCI, A&HCI, CPCI-S, CPCI-SSH, ESCI Timespan=All years | 63953 |
| 1. TS=((sensitivity analys*) or ("willingness to pay") or (quality-adjusted life year*) or (quality adjusted life year*) or (quality-adjusted life expectanc*) or (quality adjusted life expectanc*) ) Indexes=SCI-EXPANDED, SSCI, A&HCI, CPCI-S, CPCI-SSH, ESCI Timespan=All years | 467563 |
| 1. TS=utilit* Indexes=SCI-EXPANDED, SSCI, A&HCI, CPCI-S, CPCI-SSH, ESCI Timespan=All years | 396051 |
| 1. TS=(valu*) Indexes=SCI-EXPANDED, SSCI, A&HCI, CPCI-S, CPCI-SSH, ESCI Timespan=All years | 4564549 |
| 1. #4 or #5 or #6 or #7 or #8 or #9 or #10 or #11 or #12 | 8672201 |
| 1. #3 and #4 and #13 | 3804 |

CINAHL

| **Search term** | **Hits** |
| --- | --- |
| 1. MH “Economics+” | 937591 |
| 1. MH "Financial Management+" | 71012 |
| 1. MH "Financial Support+" | 600298 |
| 1. MH "Financing, Organized+" | 162919 |
| 1. MH "Business+" | 171536 |
| 1. 2 or 3 or 4 or 5 | 935931 |
| 1. 1 not 6 | 111670 |
| 1. MH "Health Resource Allocation" | 10004 |
| 1. MH "Health Resource Utilization" | 20689 |
| 1. 8 or 9 | 30117 |
| 1. 7 or 10 | 132757 |
| 1. TI (cost or costs or economic* or pharmacoeconomic* or price* or pricing*) OR AB (cost or costs or economic* or pharmacoeconomic* or price* or pricing*) | 247144 |
| 1. 11 or 12 | 326671 |
| 1. PT editorial | 323566 |
| 1. PT letter | 361775 |
| 1. PT commentary | 369669 |
| 1. 14 or 15 or 16 | 816600 |
| 1. 13 not 17 | 302970 |
| 1. MH "Animal Studies" | 138648 |
| 1. (ZT "doctoral dissertation") or (ZT "masters thesis") | 26300 |
| 1. 18 not (19 or 20) | 299343 |
| 1. Prostate | 39569 |
| 1. 21 and 22 | 1483 |

Cochrane library

| **Search term** | **Hits** |
| --- | --- |
| 1. Prostate.ti.ab.kw. | 19947 |
| 1. mri.ti.ab.kw. | 22387 |
| 1. 1 and 2 | 688 |

EconLIT

| **Search term** | **Hits** |
| --- | --- |
| 1. Prostate | 111 |

ISRCTN

| **Search term** | **Hits** |
| --- | --- |
| 1. prostate.af. | 350 |
| 1. mri.af. | 1015 |
| 1. 1 and 2 | 64 |

CRD Database

| **Search term** | **Hits** |
| --- | --- |
| 1. prostate.af. | 1057 |
| 1. mri.af. | 610 |
| 1. 1 and 2 | 27 |
