# Supplementary material for: Systematic Review and Narrative Synthesis of Economic Evaluations of Prostate Cancer Diagnostic Pathways Incorporating Prebiopsy Magnetic Resonance Imaging
Source: Eur Urol Open Sci. 2023 May 5;52:123–34. doi: 10.1016/j.euros.2023.03.010 (PMC10193166; doi:10.1016/j.euros.2023.03.010)
Supplement: Supplementary data 3 [file mmc3.docx]

| **Author** | **Year** | **Approach** | **Time Horizon** | **Perspective** | **Discounting** | **Outcome measure** | **Sensitivity analysis** |
| --- | --- | --- | --- | --- | --- | --- | --- |
| Barnett(183) | 2018 | Markov model | Lifetime from age 40 | Third-party payer | 3% | ICERs | Yes |
| Barnett(180) | 2019 | Markov model | Lifetime from age 40 | Third-party payer | 3% | ICERs | Yes |
| Brown(156) | 2018 | Decision tree + Markov model | 20 years | UK NHS | 3.5% | Cost per QALY gained at different thresholds | Yes |
| Cerantola(179) | 2016 | Markov model | 20 years | Public healthcare system | 5% | ICERs at 5-, 10- , 15- , and 20-year time horizon | Yes |
| de Rooij(182) | 2014 | Decision tree + Markov model | 10 years | Healthcare system | QALYS by 1.5%  Costs by 4% | ICERs | Yes |
| Faria(175) | 2018 | Decision tree + Markov model | Lifetime | UK NHS | 3.5% | Cost-effectiveness of diagnosis  Long-term cost-effectiveness | Yes |
| Gordon(180) | 2017 | Markov model | 30 years from age 60 | Australian government | 5% | ICERs | Yes |
| Pahwa(178) | 2017 | Decision analytic model | Lifetime | Not stated | 3% | ICERs  Net Health Benefit | Yes |

Table S1 – Model characteristics

UK – United Kingdom; NHS – National Health Service; ICERs – Incremental Cost Effectiveness Ratios; QALY – Quality Adjusted Life Year
